# Puerperal Eclampsia

**Published:** 1878-05

**Authors:** 

**Affiliations:** Oxford, Ga.


ATLANTA
Medical and Surgical Journal.

VOL. XVI.] MAY—1878. [NO. 2

### Original Communications.

### PUERPERAL ECLAMPSIA.

BY A. COUVERT, M.D., OXFORD, GA.

A year or two ago, Dr. John M. Johnson, of Atlanta, Georgia, reported, through the MEDICAL AND SURGICAL JOURNAL, a case of puerperal convulsions, which, in the absence of the family physician, a homœopathic, came into his hands for a short time, and which terminated fatally under the processes of the disciple of Hahnemann. This elicited from Dr. J. the treatment which he considered the most appropriate to such cases. I was much interested in the able and lucid article; and all the more, perhaps, as his advocacy of the lancet and its collateral measures was in such harmony with my own practice, which had yielded such satisfactory results for many years. Dr. J.'s case was brought afresh to mind by a similar one of great violence that came under my care in the city of Covington a few days ago, and which I will present in its most prominent features, avoiding the minor details that so often detract from the interest of such reports.

Mrs. B., the mother of two children, was delivered of the third child, by a negro midwife, on Friday night, 15th of March, 1878. She had had headache before and after delivery, which became so intense by Saturday night that the family physician was called in. An aperient and sinapisms abated the severity of the headache for a time.

About noon on Sunday, she fell into violent convulsions, in paroxysms of rapid succession. The family physician not being found, Dr. Alfred C. Perry, a recent and highly valued accession to the medical brotherhood of this vicinity, was called in, and with chloroform inhalations arrested the convulsions until a consultation could be had, the patient continuing in a state of profound unconsciousness from the first accession. I saw her about 3 P.M., unconvulsed, but still unconscious, presenting the usual symptoms associated with convulsions in a young, vigorous and otherwise healthy woman. I at once appealed to my old and oft-tried friend, the lancet, drew (by estimate) 24 oz. blood, and gave 20 grs. calomel, preceded by morph. hypodermically. Before leaving, we advised enemata of chloral, and brom. pot. if necessary. By the way, I would say, for the benefit of our younger brethren, rationality or even a glimmering consciousness is not necessary in administsring calomel, oil, salts, etc., if there is help enough to hold the patient in posture for a few moments. With the power of respiration and deglutition still remaining, a mouth to receive and a nose to be held, almost anything may be given to anybody of any age, without any danger of any sort, as I can testify from ample experience, running back through many years.

But to return from this digression. The effect of our first onset was so favorable that we did not apply a blister to the nape of her neck, extending down between her shoulders, as would have been best, but left, and did not see her again until 9 A.M. next day (Monday), when I learned that she had had several convulsions, though of less violence, during the night, which Dr. P. had kept in check by various judicious expedients. A blister plaster four by nine was now applied to the nape of the neck and down the spine. The calomel, retarded by the opiates, did not act until the blister began to draw, when its cathartic and revulsive effects, co-operating with the counter-irritation and depletion of the blister, cooled the fever, restored the equilibrium, awakened consciousness, and, in short, cured the patient.

Now if, in from 3,000 to 5,000 parturient patients, embracing the usual proportion of cases of eclampsia, these therapeutic measures, properly modified to suit each case, generally yields favorable results, why should we fear them, even if the lancet does usually go in front? It must not, however, be inferred that the new auxiliary remedies that science presents and experience approves should be rejected. I only urge timely and energetic treatment. What physician has not been occasionally embarrassed by the kindly intended but very injudicious interference of the friends of the patient, especially in the higher circles of society, greatly to her detriment? They shun everything that is not soothing or mild. Yet Napoleon the First, great in everything, and as exalted as any of them, told the accomplished Dubose (I think) to treat the Empress, under the impending danger, as he would a peasant. He obeyed, and saved both mother and child.

But I forbear further reflections, although written out, as they would add too much to an article already too long.

